# Hybrid spheroids containing mesenchymal stem cells promote therapeutic angiogenesis by increasing engraftment of co-transplanted endothelial colony-forming cells in vivo

**DOI:** 10.1186/s13287-023-03435-z

**Published:** 2023-08-02

**Authors:** Young Cheol Song, Gyu Tae Park, Hye Ji Moon, Eun-Bae Choi, Mi-Ju Lim, Jung Won Yoon, Nayeon Lee, Sang Mo Kwon, Byung-Joo Lee, Jae Ho Kim

**Affiliations:** 1 Department of Physiology, College of Medicine, Pusan National University, Yangsan, Gyeongsangnam-do 50612 Republic of Korea; 2 Convergence Stem Cell Research Center, Medical Research Institute, Pusan National University, Yangsan, Gyeongsangnam-do 50612 Republic of Korea; 3 Department of Otorhinolaryngology-Head and Neck Surgery, College of Medicine, Pusan National University and Biomedical Research Institute, Pusan National University Hospital, Busan, 49241 Korea

**Keywords:** Mesenchymal stem cells, Endothelial colony-forming cells, Spheroids, Peripheral artery diseases, Therapeutic angiogenesis

## Abstract

**Background:**

Peripheral artery disease is an ischemic vascular disease caused by the blockage of blood vessels supplying blood to the lower extremities. Mesenchymal stem cells (MSCs) and endothelial colony-forming cells (ECFCs) have been reported to alleviate peripheral artery disease by forming new blood vessels. However, the clinical application of MSCs and ECFCs has been impeded by their poor in vivo engraftment after cell transplantation. To augment in vivo engraftment of transplanted MSCs and ECFCs, we investigated the effects of hybrid cell spheroids, which mimic a tissue-like environment, on the therapeutic efficacy and survival of transplanted cells.

**Methods:**

The in vivo survival and angiogenic activities of the spheroids or cell suspension composed of MSCs and ECFCs were measured in a murine hindlimb ischemia model and Matrigel plug assay. In the hindlimb ischemia model, the hybrid spheroids showed enhanced therapeutic effects compared with the control groups, such as adherent cultured cells or spheroids containing either MSCs or ECFCs.

**Results:**

Spheroids from MSCs, but not from ECFCs, exhibited prolonged in vivo survival compared with adherent cultured cells, whereas hybrid spheroids composed of MSCs and ECFCs substantially increased the survival of ECFCs. Moreover, single spheroids of either MSCs or ECFCs secreted greater levels of pro-angiogenic factors than adherent cultured cells, and the hybrid spheroids of MSCs and ECFCs promoted the secretion of several pro-angiogenic factors, such as angiopoietin-2 and platelet-derived growth factor.

**Conclusion:**

These results suggest that hybrid spheroids containing MSCs can serve as carriers for cell transplantation of ECFCs which have poor in vivo engraftment efficiency.

**Supplementary Information:**

The online version contains supplementary material available at 10.1186/s13287-023-03435-z.

## Introduction

Peripheral arterial disease (PAD) is caused by the obstruction of blood vessels that supply blood to the lower extremities, such as the thighs, calves, and feet. [1]. Critical limb ischemia, a severe form of PAD, causes pain at rest, accompanied by necrosis and ulceration, leading to amputation, and has been reported to occur in up to 9.6% of those affected [2]. Patients with severe PAD can be treated with either vascular surgery or endovascular intervention that widens the narrowed blood vessels by inserting a catheter or stent into the blocked artery [3–5]. However, these treatments have critical limitations that result in temporary side effects caused by implants. Therefore, therapeutic angiogenesis, which can be induced by the delivery of proteins, genes, or cells to ischemic tissues, has drawn attention as a new strategy for PAD [6–8].

Mesenchymal stem cells (MSCs) are multipotent stem cells that can be isolated from mesodermal tissues such as the adipose, bone, placental, and tonsil tissues [9]. MSCs are promising candidates for tissue engineering and cell therapy because of their multipotent differentiation potential as well as paracrine functions [10]. They can differentiate into diverse cell types, such as adipocytes, osteoblasts, chondrocytes, and vascular cells [11]. Additionally, MSCs secrete various paracrine factors, including growth factors and pro-angiogenic cytokines, which play key roles in PAD treatment [12]. Besides MSCs, endothelial colony-forming cells (ECFCs) are also known as adult stem cells involved in angiogenesis [13]. ECFCs are generally defined as circulating cells that express endothelial cell markers, adhere to the endothelium at sites of hypoxia/ischemia, and participate in new blood vessel formation [14]. It has been reported that ECFCs are mobilized from the bone marrow into the circulation and directed to tissue sites in response to various cytokines, growth factors, and hormones that are released from damaged tissues, and they promote blood vessel formation by directly differentiating into vascular endothelial cells [15, 16]. Therefore, both MSCs and ECFCs have drawn attention as cell-based therapeutics for PAD treatment.

The poor survival of grafted cells remains an unsolved problem in cell-based therapies for ischemic diseases [17]. Reduced oxygen supply and high levels of inflammatory cytokines in ischemic tissues cause excessive production of reactive oxygen species, resulting in the death of transplanted cells [18]. It has been demonstrated that > 99% of MSCs transplanted into the uninjured heart are cleared within 5 days after cell injection [19] and > 85% of systematically injected MSCs are entrapped and lost in pre-capillaries [20]. Therefore, increasing the survival of transplanted MSCs may be crucial in improving the overall efficacy of MSC-based therapeutics. Hence, enhancing the engraftment efficiency of cell transplantation is crucial for PAD treatment. Various three-dimensional culture systems have been developed to mimic in vivo microenvironments in vitro. Spheroids, which are spherical clusters of cells formed by self-assembly, can mimic the tissue microenvironment in terms of cellular heterogeneity, nutrient and oxygen gradients, cell–cell adhesion, matrix deposition, and gene expression profiles [21]. Spheroids have been reported to enhance the in vivo viability and engraftment efficiency of transplanted cells [22–27]. For instance, MSC spheroids have been reported to enhance tissue regeneration by increasing the expression of proangiogenic factors [28, 29]. The development of functional vascular networks is crucial for tissue regeneration and angiogenesis. In vitro co-culture spheroids of vascular endothelial cells can recapitulate cell heterogeneity and the vascular system in a three-dimensional tissue microenvironment [30, 31]. The transplantation of hybrid spheroids of human MSCs and vascular endothelial cells has been reported to enhance periodontal tissue regeneration and ameliorate ischemic stroke brain injury [32, 33]. Moreover, hybrid spheroids of MSCs and ECFCs showed greater sprout numbers and cumulative sprout lengths than ECFC-only spheroids [25]. However, it is still elusive whether hybrid spherids of MSCs and ECFCs can improve therapeutic angiogenesis and engraftment of transplanted cells in vivo.

In this study, hybrid spheroids and single spheroids containing either MSCs or ECFCs were produced and the effects of the spheroids on the in vivo survival of transplanted cells and PAD therapy were observed in a murine hindlimb ischemic disease model.

## Material and methods

### Materials

Dulbecco’s Modified Eagle’s Medium (DMEM, #LM001-05), endothelial basal medium-2 (EBM-2, #CC-3156), EGM-2 MV bullet kit (#CC-3156 & CC-4147), HBSS (#H4891), Penicillin–streptomycin (#A25547), fetal bovine serum (#16000044), Trypsin–EDTA (0.5%) (#LS015-02), α-minimum essential medium (#12000022), Opti-MEM I Reduced Serum Medium (#GIB-31985–070), Calcein-AM (#C1430), Ethidium homodimer-1 (#E1169), FITC mouse anti-human CD31 antibody (#555445), FITC mouse anti-human CD34 antibody (#560942), 7-AAD staining solution (#559925), PE mouse anti-human CD166 antibody (#560903), APC mouse anti-human CD44 antibody (#BD-559942), Lipofectamine transfection reagent (#18324020) were purchased from Thermo Fisher Scientific (Waltham, MA, USA). Matrigel™ GFR basement membrane matrix (#356252), Corning® Elplasia® 6-well, Black/Clear round bottom ultra-low attachment, microcavity plate, and lid (#4440) were obtained from Corning, Inc. (Corning, NY, USA). Human recombinant VEGF 165 protein (#293-ve-010) was purchased from R&D Systems (Minneapolis, MN, USA). α-smooth muscle actin antibody (#ab5694) and human angiogenesis antibody array membrane (ab193655) were purchased from Abcam, Inc. (Cambridge, U.K.). Cryo-Gold™ cell freezing medium (#10003-01) was purchased from Revive Organtech Inc. (Irvine, CA, USA). Puromycin dihydrochloride (#P8833), 4',6-diamidino-2-phenylindole (DAPI; #D9542), and a hemoglobin assay kit (#MAK115-1KT) were purchased from Sigma-Aldrich (St. Louis, MO, USA). Dulbecco’s phosphate buffered saline (PBS; #LB001-02) was purchased from Welgene (Gyeongsan, Republic of Korea). Human basic-FGF (#100-188) was purchased from PeproTech, Inc. (Cranbury, NJ, USA). Biotinylated isolectin B4 (#B-1205-5) was purchased from Vector Laboratories (Burlingame, CA, USA).

### Flow cytometry

MSCs and ECFCs were dissociated with 0.05% trypsin‐EDTA, followed by filtration through a 40 μm cell strainer. The dissociated cells were fixed with 4% paraformaldehyde for 20 min, washed with PBS, and then incubated with APC‐conjugated anti-CD44, PE‐conjugated anti-CD166, or FITC-conjugated antibodies (CD34 and CD31) at 1:200 dilution for 20 min. The cells were washed twice, and the fluorescence intensity of the stained cells was measured using an Attune NxT flow cytometer (Thermo Fisher Scientific).

### Cell culture

MSCs were isolated from the tonsils of patients with chronic tonsillitis at the Department of Otorhinolaryngology of Pusan National University Hospital as previously reported [34]. Tonsil tissues were washed with PBS followed by the digestion in 0.075% collagenase type I (Sigma-Aldrich) at 37 °C for 30 min. After neutralization of enzyme activity with culture medium (α-modified Eagle’s medium (α-MEM) and 10% fetal bovine serum), the sample was spun down at 1000×*g* for 10 min. After centrifugation, the sample was filtered through a 50 μm nylon mesh to remove foreign matter, diluted in the culture medium, and incubated overnight at 37 °C with 5% CO_2_. The culture medium was replaced with fresh medium every 3–4 days. MSCs were positive for CD44 and CD166 and negative for CD31 and CD34 (Additional file 1: Supplementary Figure 1).

Human ECFCs were isolated from cord blood and cultured as previously described [35]. Briefly, mononuclear cells were isolated from the blood using Histopaque-1077 (Sigma-Aldrich) as previously described. Cells were seeded on culture dishes coated with rat tail collagen I (Becton Dickinson, San Jose, CA) and maintained in EBM-2 supplemented with EGM-2 MV SingleQuots containing 5% fetal bovine serum, human VEGF-1, human fibroblast growth factor-2, human epidermal growth factor, insulin-like growth factor-1, and ascorbic acid. The medium was changed 24 h after the initial plating to remove non-adherent cells and was exchanged every day for the first week. Colonies of ECFCs appeared 7–10 days after initial isolation. The cells were grown to confluence and serially passaged onto collagen I-coated dishes. ECFCs were used in passages 4–9 for all experiments, although the cells remained healthy and continued to proliferate in passages 10 and above. The expression profiles of cell surface markers in ECFCs were examined using flow cytometry. ECFCs expressed ECFC-specific cell surface markers (CD34 and CD31), but not MSC markers (CD166 and CD44; Additional file 1: Fig. S1). To culture Hybrid^2D^, MSCs and ECFCs were seeded onto a cell culture dish and cultured with a mixture of MSC and EC culture media (50:50).

### Spheroid culture

Ultra-low attachment 96-well plates (Corning® Elplasia® plates) were used for 3D spheroid cell culture. MSCs and cord blood-derived ECFCs were seeded onto a hyperplasia plate to produce MSC^3D^ and ECFC^3D^, respectively. To establish Hybrid^3D^, MSCs and ECFCs were co-cultured on a hyperplasia plate. The 2D-cultured MSCs and ECFCs were dissociated into a single cell by treatment with trypsin, and cells were seeded at a density (500 cells/well) into 96-well plates (100 μL/well in each well of a 96-well plate) in EBM-2 medium containing 20% fetal bovine serum (Thermo Fisher Scientific). After incubation at 37 °C and 5% CO_2_ for 24 h, spheroids were collected from the plates. The spheroid diameter was measured using EVOS cell imaging system (Thermo Fisher Scientific).

### Preparation of conditioned medium

To obtain a conditioned medium, the cells were cultured and seeded at a density of 10,000 cells/cm^2^. At 80% confluence, the cells were washed three times with PBS and the medium was replaced with a serum-free medium. After 24 h, the media were centrifuged (Eppendorf, Hauppauge, NY, USA) at 300×*g* for 5 min, filtered through a 0.22 μm filter (Pall Corporation, Port Washington, NY, USA), and were then stored at − 70 °C until use.

### Lentivirus infection

To produce lentiviruses expressing GFP and mCherry genes, GFP (pLVX-AcGFP-C1) and mCherry plasmids (pCDH-CMV-mCherry-T2A-Puro) were purchased from Addgene (www.addgene.org). 293FT cells were co-transfected with the lentiviral vector along with the packaging plasmids (Delta 8.9, and VSV-G) using Lipofectamine-Plus reagent. 293FT cells were maintained in DMEM containing 10% fetal bovine serum. Forty-eight hours after transfection, the virus-containing supernatant was collected, passed through a 0.45 μm filter, and stored at − 80 °C. For lentiviral transduction, MSC and ECFC were treated with cell culture supernatant containing lentivirus and 10 μg/mL polybrene (Sigma-Aldrich) to produce mCherry-expressing MSCs and GFP-expressing ECFCs. The fluid cytometric analysis confirmed GFP and mCherry expressions in the ECFCs and MSCs, respectively (Additional file 1: Supplementary Figure 2).

### Mouse hindlimb ischemia model

6-week-old male BALB/CA-nu/nu mice, with an average weight of 20–24 g, were purchased from Orient Bio (Seongnam-si, Korea). All animals were bred in an air-conditioned animal room with relative humidity and fed laboratory feed and water. After the mice were anesthetized with an intraperitoneal injection of 400 mg/kg 2,2,2 tribromoethanol (Avertin; Sigma-Aldrich), the femoral artery was resected from its proximal origin at the branch of the external iliac artery to the distal point, where it bifurcates into the saphenous and popliteal arteries. After arterial ligation, the mice were randomly divided in a double-blind manner into seven groups including control group (n = 5/each group), followed by injection of PBS (control group), single dissociated cells or spheroids of MSCs and ECFCs. The total number of transplanted cells was 1 × 10^6^ cells per mouse and the cells were injected into four sites (20 μL at each site) of the gracilis muscle in the medial thigh. Blood flow was measured at 0, 7, 14, 21, and 28 days after hindlimb induction using a laser Doppler perfusion imaging (LDPI) analyzer (Moor Instruments Ltd., Devon, UK). Perfusion of ischemic and non-ischemic limbs was calculated based on the pixels of the color histogram. Red and blue colors indicate high and low perfusion levels, respectively. Hemoperfusion is expressed as the LDPI index and ischemic to non-ischemic limb blood flow ratio. A preoperative ratio of 1 indicates equal blood perfusion in both feet. The degree of ischemic hindlimb necrosis was recorded 28 days after the surgery. The necrosis score was evaluated as follows: 0, limb recall; 1, toe amputation; 2, foot amputation; and 3, limb amputation.

### Matrigel plug assay

A Matrigel plug assay was performed to evaluate the in vivo angiogenic potential of the cells. Briefly, BALB/C nude mice were anesthetized and subcutaneously injected with 500 μL of growth factor-reduced Matrigel containing vascular endothelial growth factor (VEGF), the 2D- or 3D-cultured cells (2 × 10^5^ cells/ECFCs, MSCs, and hybrid cells). The Matrigel plugs were excised after 2 weeks and stained with hematoxylin and eosin (H&E) staining to observe the formation of new blood vessels. We also determined the hemoglobin content in the Matrigel plugs by using a hemoglobin assay kit. Matrigel plugs were homogenized in a water-heparin solution and centrifuged at 1500×*g* for 15 min at 20 °C. Hemoglobin content of the supernatant (100 μL) was determined using Drabkin’s method [36]] at 540 nm using spectrophotometry.

### Immunohistochemistry

For immunohistochemical analysis of tissue specimens, hindlimb muscles were removed, formalin-fixed, and freeze-embedded with Optimal cutting temperature compound. Frozen specimens were cut into 10 μm thick sections, stained with H&E solution, and photographed under a microscope (Axioimager M2, Carl Zeiss, Heidenheim, Germany). Endothelial and smooth muscle cells were immunostained with rabbit ILB4 and anti-α-SMA antibodies, respectively. Specimens were incubated with Alexa488 goat anti-rabbit or Alexa568 mouse anti-secondary antibody, washed, and mounted onto glass slides with Vectashield medium (Vector Laboratories, Burlingame, CA) containing DAPI for nuclear visualization. Stained images were acquired with a laser-scanning confocal microscope (Carl Zeiss AG, Oberkochen, Germany, LSM 900).

### In vivo fluorescence imaging of transplanted cells

To measure the in vivo cell engraftment and survival of MSC-mCherry and ECFC-GFP, the cells were diluted with Matrigel solution (500 μL/mouse) and subcutaneously injected into the dorsal side of mice using a 24-gauge Hamilton syringe. To measure the fluorescence of the transplanted cells, mice were visualized with an in vivo imaging system (CRi Maestro, Cambridge Research & Instrument, Inc., Hopkinton, MA) 1 day, 1 week, 2 weeks, and 3 weeks after cell transplantation.

### Analysis of endothelial tube formation

To determine the endothelial tube-forming ability of ECFCs, growth factor-reduced Matrigel was added to 48-well culture plates and polymerized for 30 min at 37 °C. ECFCs (4 × 10^4^ cells) were seeded on Matrigel-coated plates and cultured in EBM-2 medium with 10 ng/ml VEGF or conditioned medium collected from the ECFCs, MSCs, and hybrid cells. After 24 h, cells were fixed with 4% paraformaldehyde and visualized using an EVO SM5000 imaging system.

### Cell migration

ECFC migration was assayed using a disposable 96-well chemotactic chamber (Neuro Probe, Inc., Gaithersburg, MD, USA), according to the manufacturer’s instructions. Briefly, 2D-cultured ECFCs were dissociated by treatment with 0.05% trypsin containing 0.02% EDTA, washed once, and suspended in EBM-2 at a concentration of 2 × 10^5^ cells/mL. Membrane filters (8 μm pore size) in disposable 96-well chemotaxis chambers were incubated with 20 mg/ml rat-tail collagen overnight at room temperature for collagen coating. Aliquots (50 μL per well) of the cell suspension were loaded into the upper chambers, and test reagents were placed in the lower chamber. Following incubation for 12 h at 37 °C, the upper surface of each filter was scraped free of cells by wiping it with a cotton swab. The number of cells that migrated to the lower surfaces of each filter was determined by microscopically counting the cells in four places (×100 magnification) after fixation with 4% paraformaldehyde and staining with 0.1% Hoechst 33,342 staining solution (Thermo Fisher Scientific).

### Analysis of cytokine profiles with a protein array

Cytokine profiles were evaluated using a Proteome Profiler Human XL Cytokine Array Kit (R&D Systems). The experimental procedure for measuring cytokine levels in the cell-conditioned medium, which were collected from ECFC^2D^, ECFC^3D^, MSC^2D^, MSC^3D^, Hybrid^2D^, and Hybrid^3D^, was performed according to the manufacturer's instructions.

### Statistical analysis

The results of multiple observations are presented as mean ± SD. For multivariate data analysis, group differences were assessed using one-way or two-way analysis of variance (ANOVA). To ensure whether the data met the assumptions of the statistical approach, we checked for normality using the Shapiro–Wilk test, homogeneity of variance using Levene’s test, and independence of observations.

## Results

### Fabrication of hybrid spheroids composed of MSCs and ECFCs by 3D suspension culture

**Fig. 1:**
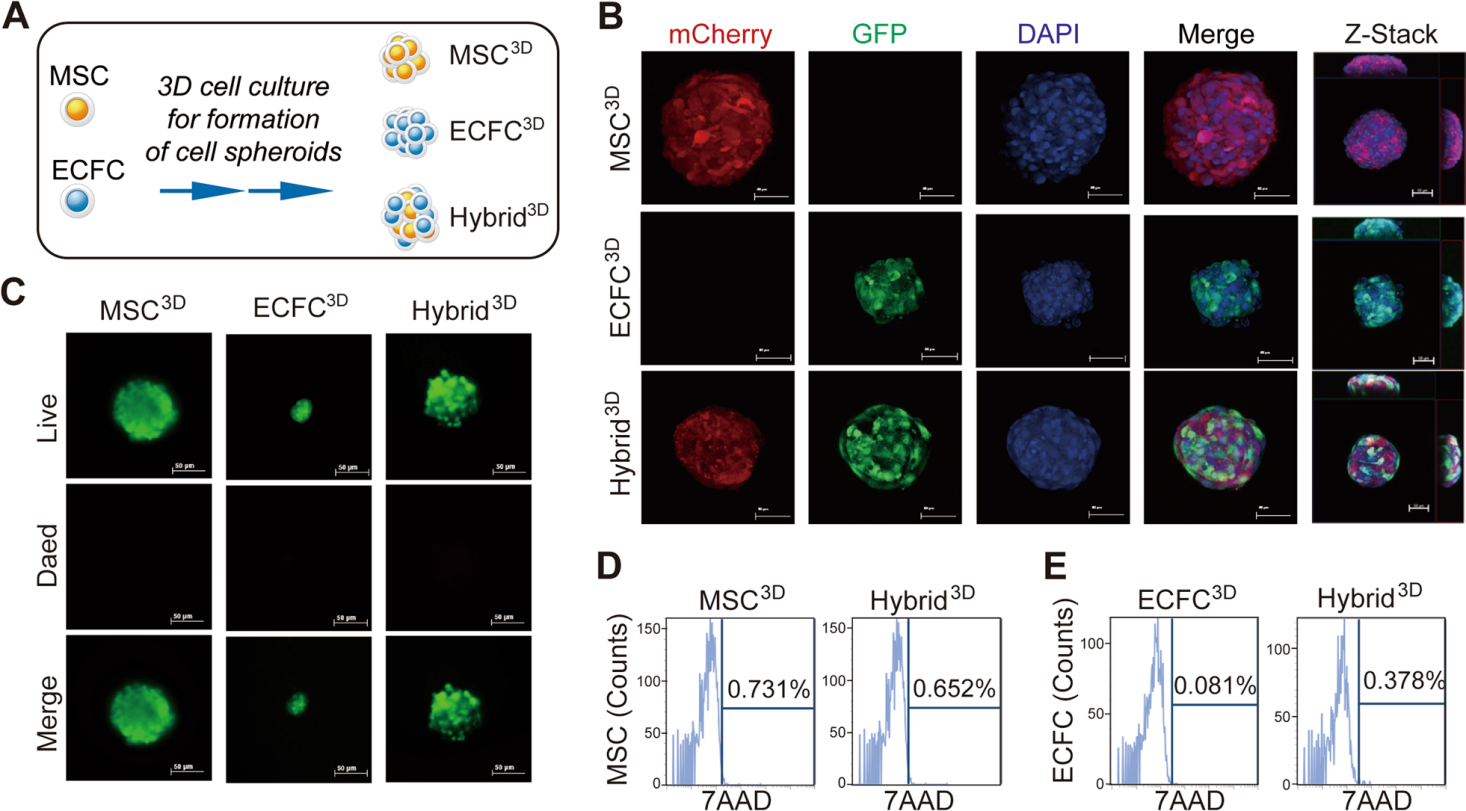
Formation of MSC, ECFC, Hybrid spheroids by 3D culture of MSCs and ECFCs. **A** Schematic diagram for the generation of MSC^3D^, ECFC^3D^, and hybrid^3D^ from MSC and ECFC. **B** Fluorescence microcopy images of MSC-mCherry and ECFC-GFP in MSC^3D^, ECFC^3D^, and hybrid^3D^. Nuclei were counterstained with DAPI (blue), and merged images with mCherry (red) and GFP (green) colors are shown. Side view (view in the *x* and *z* plane) and bottom view (view in the *y* and *z* plane) of the z-stack images are shown. Scale bar = 50 μm. **C** Staining of live and dead cells in MSC^3D^, ECFC^3D^, and hybrid^3D^. Cell spheroids were stained with a live/dead cell staining kit, and fluorescence images of live cells (green), dead cells (red), merged images are shown. **D** FACS analysis of 7AAD-positive dead cells in MSC^3D^ and hybrid^3D^. The population of 7AAD-and CD166-double positive cells in the MSC^3D^ and hybrid^3D^ was measured using FACS analysis. **E** FACS analysis of 7AAD-positive dead cells in ECFC^3D^ and hybrid^3D^. The population of 7AAD-and CD34-double positive cells in the ECFC^3D^ and hybrid^3D^ was measured using FACS analysis

In this study, we used tonsil-derived MSCs and cord blood-derived ECFCs for cell therapy. The tonsil-derived MSCs have been shown to exhibit superior proliferation potential than the other tissue-derived MSCs [37]], suggesting that tonsil-derived MSCs are promising sources for the large-scale manufacturing of therapeutics and tissue engineering. To assemble cells into 3D spheroids, cell suspensions of MSC-mCherry and ECFC-GFP were added to a 12-well ultra-low attachment dish. After 24 h of culture, MSCs and ECFCs were condensed into spherical aggregates, MSC spheroids (MSC^3D^) and ECFC spheroids (ECFC^3D^), respectively. Co-culture of MSCs and ECFCs in the ultra-low attachment dish forms mixed spheroids (Hybrid^3D^) which contain both MSCs and ECFCs (Fig. 1A). Next, we assessed the distribution of each cell type within 3D spheroids using confocal microscopy. MSC^3D^ expressed mCherry, but not GFP. ECFC^3D^ expressed GFP but not mCherry (Fig. 1B). Hybrid^3D^ generated fluorescence signals positive for both mCherry and GFP. Z-stack confocal images of the fabricated Hybrid^3D^ indicated the incorporation of both cell types into the spheroids. The average diameters of MSC^3D^, ECFC^3D^, and Hybrid^3D^ were estimated to be 150 ± 10, 100 ± 10, and 125 ± 10 μm, respectively. The diameter of ECFC^3D^ was observed to be smaller than that of MSC^3D^, which is due to the smaller size of ECFCs compared to MSCs.

The viability of cells in MSC^3D^, ECFC^3D^, and Hybrid^3D^ was measured by staining with a live/dead cell™ imaging kit. Live cells stained by Calcein-AM emits green fluorescence, whereas the s dead cells stained by membrane-impermeable DNA dye ethidium homodimer-1 emits red fluorescence. As shown in Fig. 1C, most cells exhibited a green color, whereas red colored dead cells were hardly detected. To confirm these results, MSC^3D^, ECFC^3D^, and Hybrid^3D^ were dissociated into single cells, stained with 7-AAD (7-aminoactinomycin D), and subjected to flow cytometry analysis to detect dead cells. The percentages of 7-AAD-positive MSCs or ECFCs in MSC^3D^, ECFC^3D^, and Hybrid^3D^ were under 1% (Figs. 1D and E). These results suggest that MSCs and ECFCs in spheroids exhibit good viability for cell therapy.

### Transplantation of Hybrid^3D^ stimulates blood perfusion and alleviates tissue necrosis in hindlimb ischemia

**Fig. 2:**
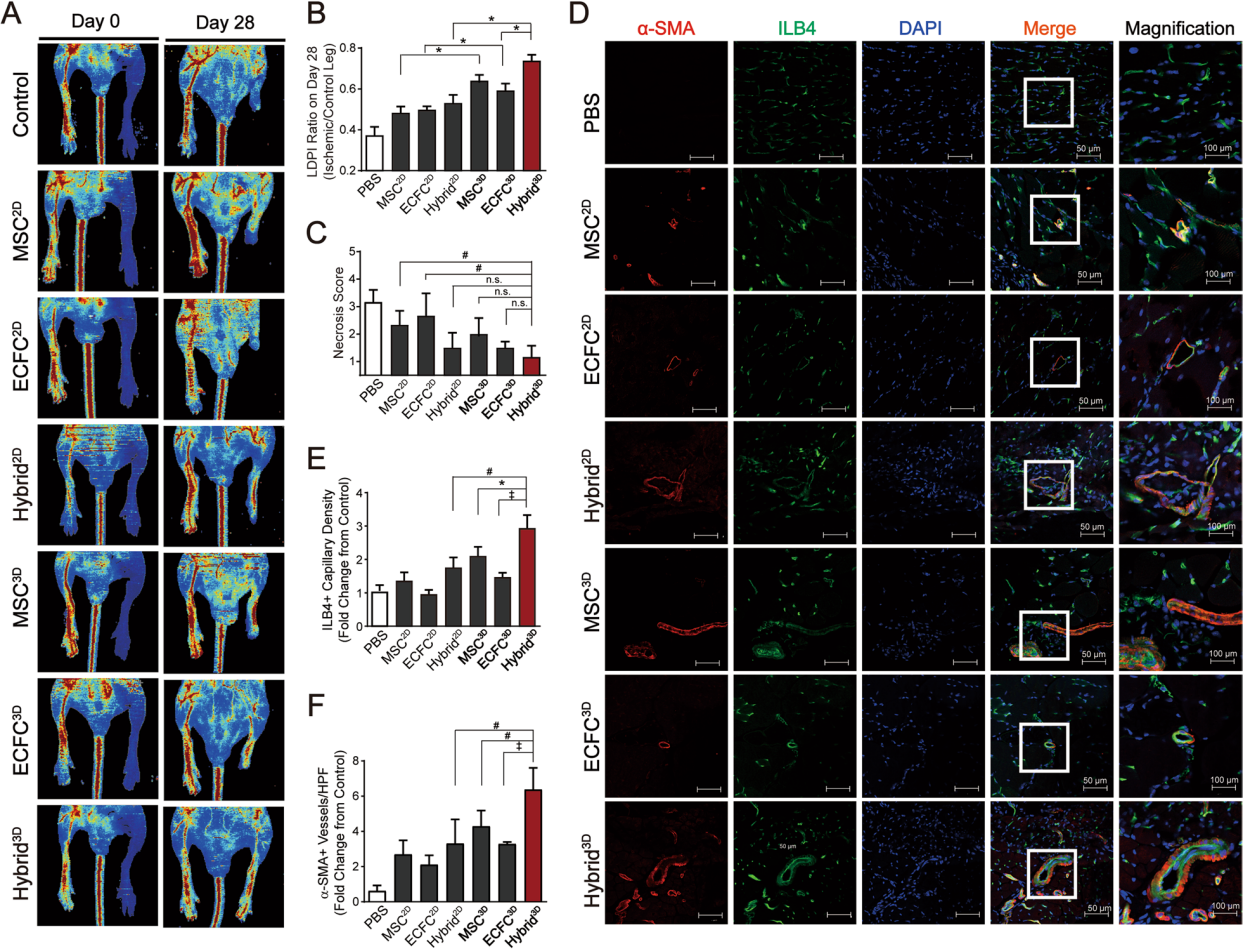
Therapeutic effects of Hybrid^3D^ transplantation in the hindlimb ischemia models. **A** Representative photographs and laser Doppler perfusion imaging (LDPI) of mouse hindlimbs on day 0 and 28 after transplantation of MSC^2D^, ECFC^2D^, Hybrid^2D^, MSC^3D^, ECFC^3D^, and Hybrid^3D^ (n = 5). **B** Quantitative analysis of the blood perfusion recovery measured using an LDPI analyzer. The LDPI ratio was calculated as the ratio of ischemic to nonischemic hindlimb blood perfusion on day 28. **C** Statistical analysis of the necrosis score on day 28. **D** Immunostaining of α-SMA^+^ blood vessels (red) and ILB4^+^ capillaries (green) in ischemic limbs at 28 days after surgery. Nuclei (blue) were counterstained with DAPI and merged images are shown. White boxes are the regions magnified. Quantification of ILB4^+^ capillaries (**E**) and α-SMA^+^ (**F**) arteries in the ischemic limb by immunohistochemistry. Results are presented as mean ± SD. ^*^p < 0.05, ^#^*p* < 0.01, ^‡^*p* < 0.001, n.s.: not significant

The therapeutic effects of Hybrid^3D^ were measured in a murine hindlimb ischemia model. After inducing hindlimb ischemia, two-dimensional cultured MSC (MSC^2D^) and ECFC (ECFC^2D^) were dissociated into a single cell suspension, followed by intramuscular injection of MSC^2D^, ECFC^2D^, and the mixture of MSC^2D^ and ECFC^2D^ (Hybrid^2D^) into the ischemic limbs. Moreover, MSC^3D^, ECFC^3D^, and Hybrid^3D^ were injected into the ischemic limbs. Blood flow in the ischemic and non-ischemic limbs was measured using LDPI (Fig. 2A), and the LDPI ratio of the ischemic limb to non-ischemic limb at day 28 was calculated. Blood flow in the ischemic limbs was significantly enhanced by transplantation of MSC^2D^, ECFC^2D^, or Hybrid^2D^. Moreover, the blood flow in the ischemic limbs transplanted with MSC^3D^ or ECFC^3D^ was significantly greater than those transplanted with MSC^2D^ or ECFC^2D^ (Fig. 2B). Furthermore, Hybrid^3D^ transplantation further increased blood flow compared with the transplantation of Hybrid^2D^, and tissue necrosis was substantially reduced in ischemic limbs transplanted with Hybrid^2D^, MSC^3D^, ECFC^3D^, or Hybrid^3D^ (Fig. 2C).

Angiogenesis is crucial for ischemic tissue repair and recovery of blood perfusion [38]. To assess whether Hybrid^3D^ can stimulate angiogenesis in vivo, the blood vessel density in ischemic limbs was measured by immunostaining. The densities of capillary blood vessels and arteries/arterioles in ischemic limbs were measured by staining of ILB4 and α-SMA, respectively (Fig. 2D). The numbers of ILB4-positive capillaries and α-SMA-positive arteries/arterioles in ischemic limbs transplanted with either MSC^3D^ or ECFC^3D^ were slightly greater than those in limbs transplanted with either MSC^2D^ or ECFC^2D^ (Fig. 2E and F). Notably, intramuscular transplantation of Hybrid^3D^ significantly increased the number of ILB4-positive capillaries and α-SMA-positive arteries/arterioles in ischemic limbs compared to MSC^3D^, ECFC^3D^, or Hybrid^2D^ (Fig. 2E and F). These results suggest that the angiogenic activities of Hybrid^3D^ are greater than those of MSC and ECFC single-cell spheroids.

### Increased in vivo survival of MSCs and ECFCs in the Hybrid^3D^-transplanted Mice

**Fig. 3:**
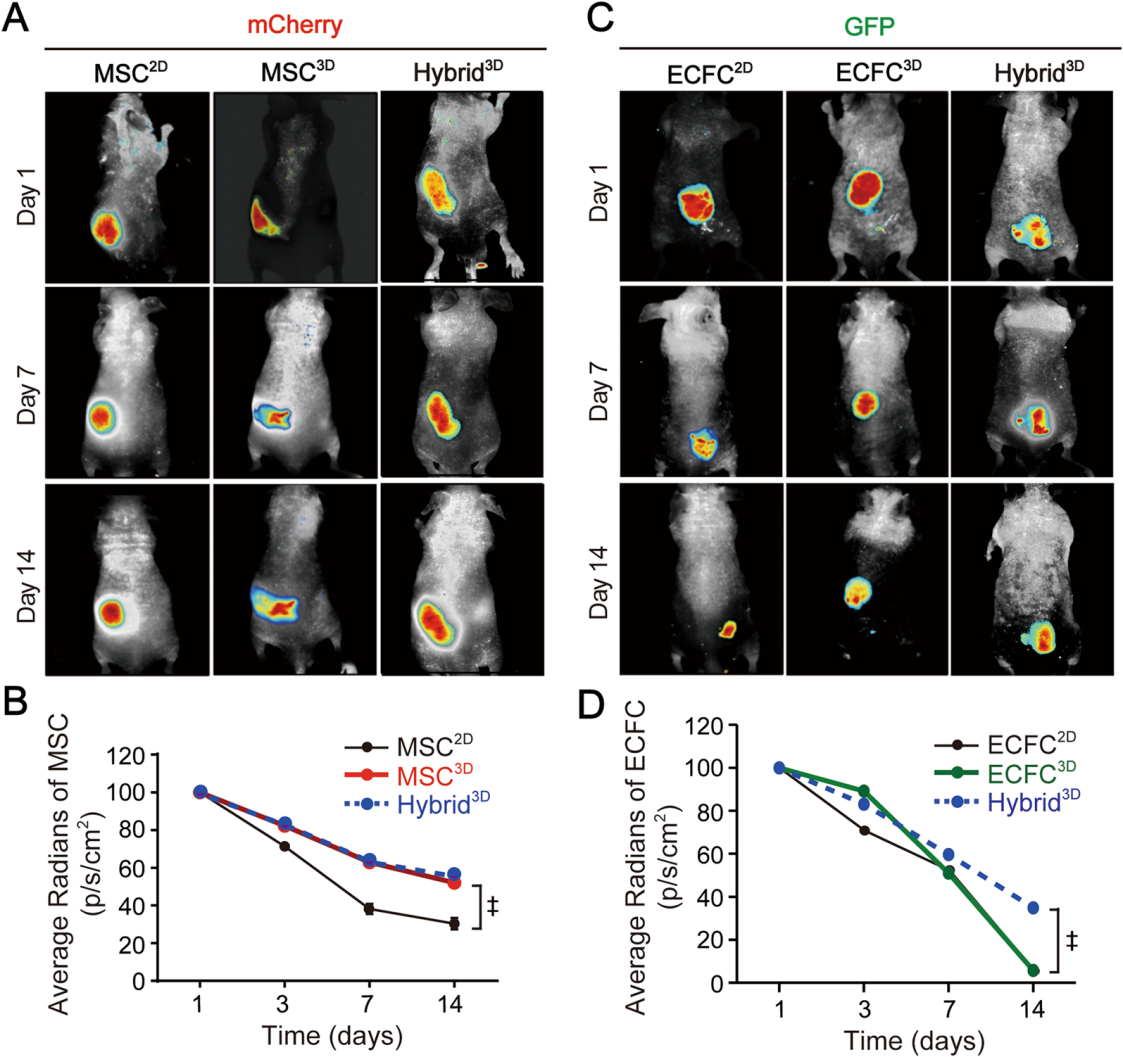
Enhanced in vivo survival of MSCs and ECFCs in mice transplanted with Hybrid^3D^. **A** Effects of 3D spheroid cell culture on in vivo survival of MSCs. MSC^2D^, MSC^3D^, or Hybrid^3D^ were subcutaneously transplanted onto the back of skin flaps of athymic mice, and the fluorescence of MSC-mCherry in MSC^2D^, MSC^3D^, and Hybrid^3D^ were measured using CRi Maestro in vivo imaging system on 1, 7, 14 days after cell transplantation. **B** In vivo fluorescence image analysis was performed on days 1, 3, 7 and 14 to determine the survival rates of MSC-mCherry in in MSC^2D^, MSC^3D^, and Hybrid^3D^. Results are presented as mean ± SD. ^‡^*p* < 0.001, MSC^2D^ vs. MSC^3D^ and Hybrid^3D^. **C** Effects of 3D spheroid cell culture on in vivo survival of ECFCs. ECFC^2D^, ECFC^3D^, or Hybrid^3D^ were subcutaneously transplanted onto the back of skin flaps of athymic mice, and the fluorescence of ECFC-GFP in ECFC^2D^, ECFC^3D^, or Hybrid^3D^ were measured on 1, 7, 14 days after cell transplantation. **D** In vivo fluorescence image analysis was performed on days 1, 3, 7 and 14 to determine the survival rates of ECFC-GFP in in ECFC^2D^, ECFC^3D^, or Hybrid^3D^. Results are presented as mean ± SD. ^‡^*p* < 0.001, Hybrid^3D^ vs. ECFC^2D^ and ECFC^3D^

To explore whether prolonged in vivo survival is implicated in enhanced angiogenesis and tissue repair in the Hybrid^3D^-transplanted limbs, two-dimensional cultured cells and spheroids composed of mCherry-expressing MSCs and GFP-expressing ECFCs were subcutaneously injected into BALB/c nude mice. The fluorescence of the transplanted cells was measured at 1, 7, and 14 days after cell transplantation using an in vivo imaging system. The fluorescence of transplanted MSC^2D^ cells decreased in a time-dependent manner to approximately 40% of the initial fluorescence on day 14 (Fig. 3A and B). However, the fluorescence of MSC^3D^ reduced slowly compared with that of MSC^2D^ and reached approximately 60% on day 14, and there was no significant difference between the MSC^3D^- and Hybrid^3D^-injected groups on day 14 (Fig. 3B). The fluorescence of ECFC^2D^ and ECFC^3D^ decreased in a time-dependent manner. However, there was no significant difference between the two groups on day 14 (Fis. 3C and D). Notably, the fluorescence of ECFC in Hybrid^3D^ was significantly higher than that of ECFC^3D^ on day 14 (Fig. 3D). These results suggest that MSC spheroids exhibit prolonged in vivo survival and that hybrid spheroids composed of MSCs and ECFCs are highly useful as cell therapeutics because of the enhanced survival of not only MSCs but also ECFCs.

### Transplantation of Hybrid^3D^ stimulates angiogenesis in vivo

**Fig. 4:**
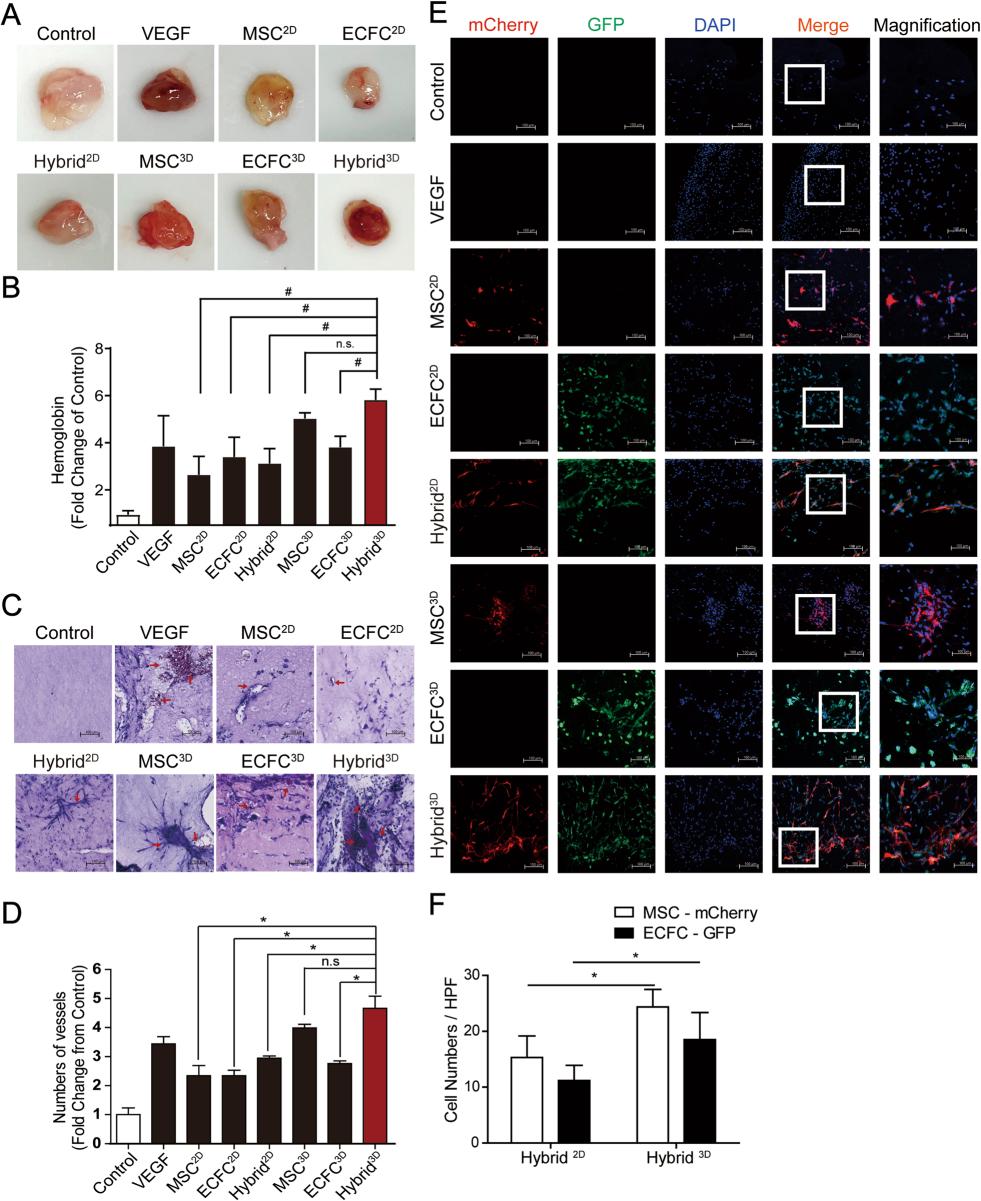
Effects of spheroid cell culture on in vivo angiogenesis. **A** Measurement of in vivo angiogenesis using Matrigel plug assay after 3D cell spheroids. Athymic nude mice were subcutaneously injected with 500 μL of growth factor-reduced Matrigel containing recombinant VEGF protein (10 ng/mL), 2D cultured cells, or 3D spheroids. Gross images of Matrigel plugs harvested 2 weeks after subcutaneous injection into athymic nude mice. **B** Quantification of hemoglobin concentration in each Matrigel plug. **C** Representative H&E-stained images of Matrigel plugs; arrows indicate vascular structure containing red blood cells. Scale bar = 100 μm. **D** Quantification of the numbers of blood vessels in the H&E-stained images of Matrigel plugs. **E** Immunofluorescence staining of MSC-mCherry and ECFC-GFP in the Matrigel plug tissues. Nuclei were stained with DAPI and overlaid **F** Quantification of the cell numbers of MSC-mCherry and ECFC-GFP in Matrigel plugs containing Hybrid^2D^ and Hybrid^3D^. Data are expressed as mean ± SD (n = 6). *p < 0.05; ^#^*p* < 0.01; n.s.: not significant

To determine the pro-angiogenic activities of Hybrid^3D^ in vivo, we next examined the angiogenic potentials of 2D-cultured cells and spheroids composed of mCherry-expressing MSCs and GFP-expressing ECFCs using a Matrigel plug assay. Single cells or spheroids were resuspended in Matrigel solution and injected subcutaneously into BALB/c nude mice, and angiogenesis in the Matrigel plugs was evaluated 14 days after cell transplantation using the hemoglobin assay and H&E staining. In the in vivo Matrigel plug assay, the amount of hemoglobin in the Matrigel plugs was increased by the inclusion of VEGF, MSC^2D^, ECFC^2D^, Hybrid^2D^, MSC^3D^, or ECFC^3D^ compared with that in the control group (Fig. 4A and B). Specifically, the hemoglobin value of MSC^3D^ increased twofold compared to that of MSC^2D^, and the hemoglobin value of ECFC^3D^ increased 1.5-fold compared to that of ECFC^2D^ (Fig. 4C). The hemoglobin levels of the Hybrid^3D^-containing Matrigel plugs were greater than those of the plugs containing MSC^2D^, ECFC^2D^, Hybrid^2D^, or ECFC^3D^, suggesting increased blood vessel formation in the Hybrid^3D^-transplanted Matrigel plugs. Additionally, sections of the Matrigel plugs were stained with H&E to measure new blood vessels containing red blood cells. The cell-embedded Matrigel plugs had a higher number of blood vessels containing red blood cells than the control plugs (Fig. 4C and D). Moreover, the number of blood vessels of the Hybrid^3D^-containing Matrigel plugs was greater than that of the plugs containing Hybrid^2D^.

We next examined the distribution of transplanted mCherry-expressing MSCs and GFP-expressing ECFCs within the Matrigel plugs using confocal microscopy (Figs. 4E). Matrigel plugs containing MSCs or ECFCs exhibited mCherry- and GFP-positive signals, respectively. Moreover, both Hybrid^2D^ and Hybrid^3D^ showed the co-localization of mCherry- and GFP-positive cells within Matrigel plugs, while Hybrid^3D^ exhibited a greater number of MSCs and ECFCs than Hybrid^2D^ (Fig. 4F). These results suggest that Hybrid^3D^ promotes angiogenesis in vivo by increasing the survival of the transplanted cells.

### Hybrid^3D^ enhances angiogenesis in vitro through a paracrine-dependent mechanism

**Fig. 5:**
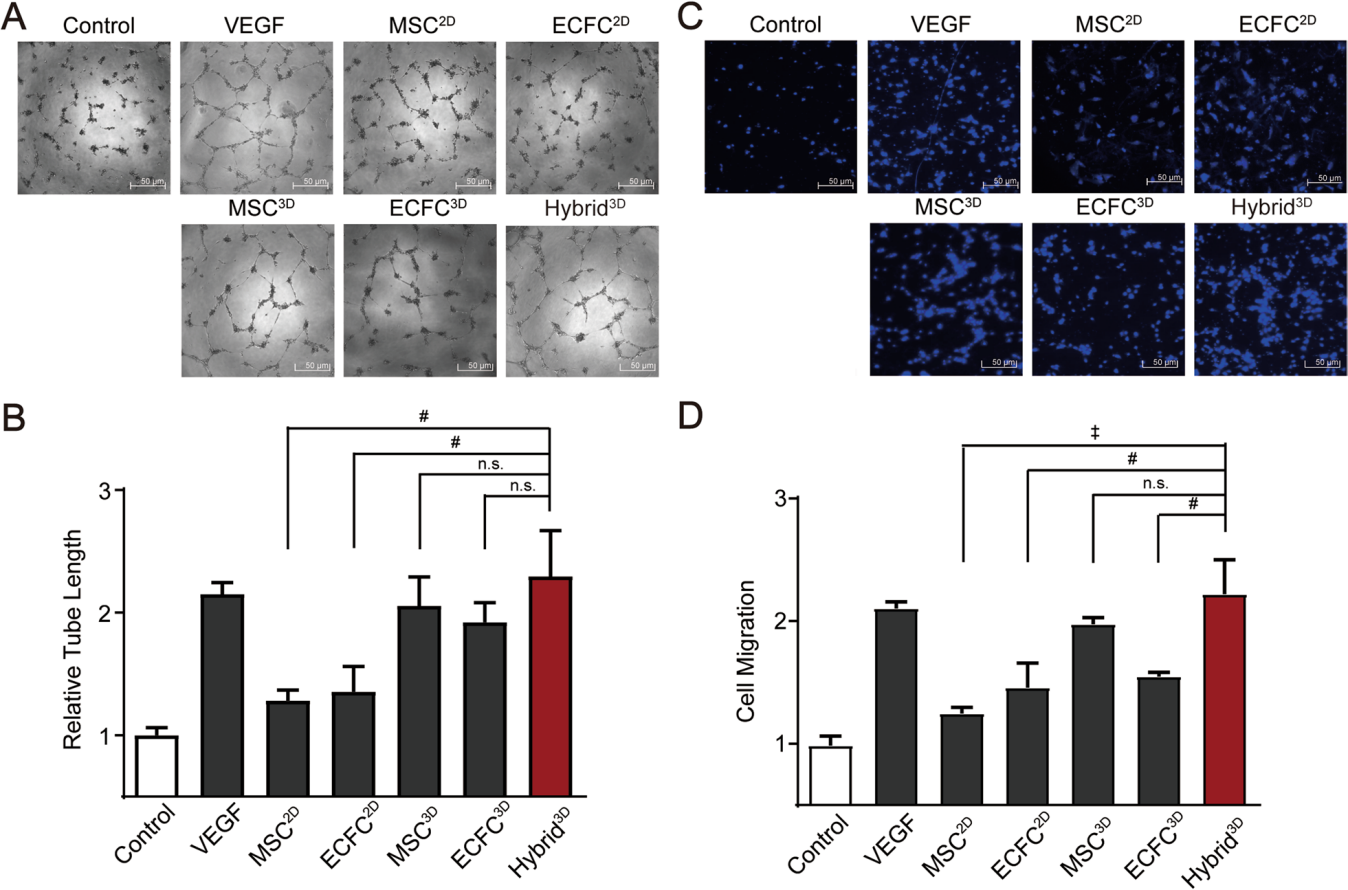
Paracrine stimulation of angiogenesis by Hybrid^3D^ spheroids. **A** Endothelial tube forming assay. ECFCs were treated with recombinant VEGF, the conditioned medium derived from the 2D cultured cells or 3D spheroids for 2 days. The cells were photographed by phase microscopy. Scale bar = 50 μm. **B** In vitro endothelial tube length was measured using ImageJ. **C** Effects of cell spheroid-conditioned medium on ECFC migration. ECFC migration was measured using disposable 96-well chemotactic chamber. The migrated cells were stained with Hoechst 33,342 staining solution. Scale bar = 50 μm. **D** Cell migration was measured by quantifying the numbers of nuclei. Data are expressed as mean ± SD (n = 4). ^#^*p* < 0.01, ^‡^*p* < 0.001, n.s.: not significant

To investigate the molecular mechanisms underlying Hybrid^3D^-stimulated angiogenesis, conditioned media were collected from the 2D- and 3D-cultured cells and the effects of the conditioned media on the endothelial tube-forming capacity of ECFCs were measured. The endothelial tube-forming ability of ECFCs was more potently stimulated by the conditioned medium collected from the cultures of MSC^3D^, ECFC^3D^, and Hybrid^3D^ compared to that of MSC^2D^ and ECFC^2D^ (Fig. 5A and B). Next, we performed a transwell migration assay to measure the pro-angiogenic ability of MSC^3D^-, ECFC^3D^, and Hybrid^3D^. The endothelial migration ability could be augmented by treatment with the conditioned medium obtained from the culture of MSC^3D^ and Hybrid^3D^ (Fig. 5C and D), and they had more potent effects on endothelial migration than the conditioned medium from ECFC^3D^. The conditioned medium from Hybrid^3D^ stimulated endothelial tube formation and migration as potently as VEGF (Fig. 5B and D). These results suggest that Hybrid^3D^ promotes angiogenesis stimulation through a paracrine-dependent mechanism.

### Analysis of pro-angiogenic factors secreted from MSCs and ECFCs

**Fig. 6:**
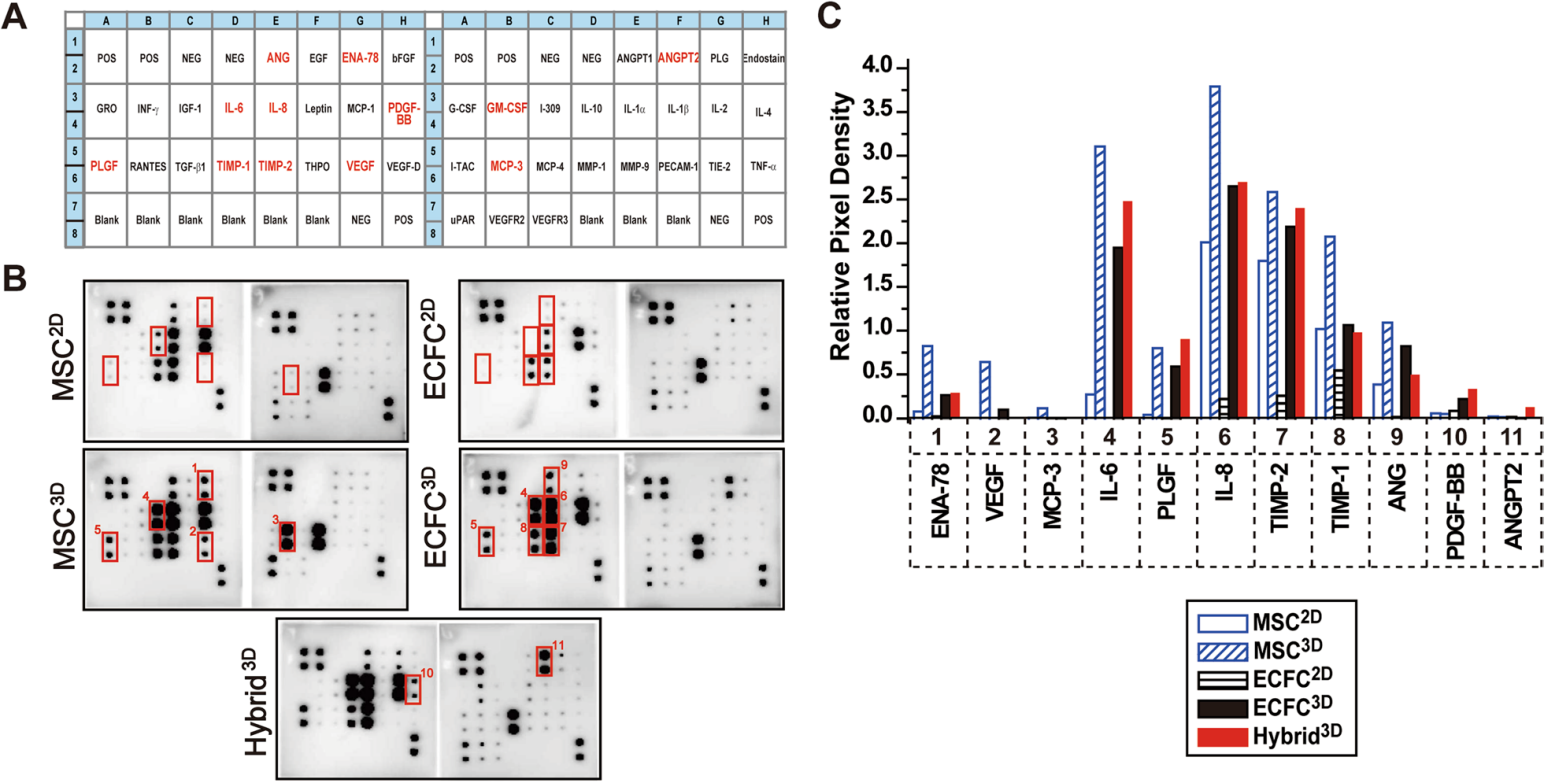
Analysis of pro-angiogenic factors secreted from MSCs and ECFCs. **A** Angiogenesis antibody array maps used in this study. The red color indicates cytokines secreted from 3D spheroids. **B** Angiogenesis antibody array of conditioned medium derived from MSC^2D^, ECFC^2D^, MSC^3D^, ECFC^3D^, and Hybrid^3D^. A membrane-based antibody array was used to determine human angiogenesis-related proteins in conditioned medium derived from MSC^2D^, ECFC^2D^, MSC^3D^, ECFC^3D^, and Hybrid^3D^. The red boxes indicate protein spots which are significantly different between MSC^3D^ vs. MSC^2D^, ECFC^3D^ vs. ECFC^2D^, and Hybrid^3D^ vs. MSC^3D^ and ECFC^3D^. Representative data from two independent experiments are shown. **C** The pixel densities of the twelve red colored boxes in panel B were quantified using Image J and normalized to reference spots on the membranes

To investigate the paracrine mechanisms underlying Hybrid^3D^-mediated angiogenesis, we performed angiogenic protein arrays of conditioned medium obtained from 2 and 3D cultures of MSCs and ECFCs. The protein array includes 43 pro-angiogenic proteins (Fig. 6A). We compared MSC^2D^ and MSC^3D^ and found that MSC^3D^ secreted increased levels of angiogenesis-related factors, including ENA-78, VEGF, MCP-3, IL-6, and placental growth factor (PlGF) (Fig. 6B and C). In addition, compared to ECFC^2D^, ECFC^3D^ secreted higher IL-6, PlGF, IL-8, TIMP-2, TIMP-1, and ANG levels (Fig. 6B and C). Moreover, Hybrid^3D^ secreted higher PDGF-BB and ANGPT2 levels than MSC^3D^ and ECFC^3D^ (Fig. 6C). These results suggest that spheroid culture of MSCs and ECFCs stimulates the secretion of pro-angiogenic factors, and 3D co-culture of MSCs and ECFCs affects the secretion of pro-angiogenic factors by stimulating cell–cell communication.

## Discussion

Stem cells have drawn attention as a new treatment for PADs and critical limb ischemia [17]; however, their clinical application has been hampered by the poor survival of transplanted cells in vivo. In this study, we demonstrated that MSC^3D^, but not ECFC^3D^, exhibits increased in vivo survival of transplanted cells. Furthermore, Hybrid^3D^ spheroids, which were generated by 3D co-culture of MSCs and ECFCs, augmented the in vivo survival of not only MSCs but also ECFCs, suggesting that the hybrid spheroids containing MSCs enhance the in vivo engraftment of co-transplanted ECFCs. After in vivo transplantation, the number of ECFCs in Hybrid^3D^ was significantly higher than that in ECFC^3D^, but the numbers of MSCs in Hybrid^3D^ and MSC^3D^ were not significantly different. These results suggest that Hybrid^3D^ stimulates survival and proliferation of ECFCs in vivo. MSC spheroids have been shown to have improved survival in vivo compared to single-cell suspensions of MSCs [22], despite MSC^3D^ having a lesser survival advantage than MSC^2D^ in vitro [39]. It has been reported that the anti-apoptotic protein Bcl-2 is upregulated whereas the proapoptotic protein Bax is downregulated, thereby increasing the survival of spheroidal cells [22]. Spheroid formation enhances the survival and stemness of transplanted MSCs and delays in vitro replicative senescence [40]. Intramuscular injection of adipose-derived MSC spheroids showed better proliferation than MSC suspension in the ischemic region, as evidenced by increased expression of the proliferation marker PCNA [41]. Various clinical trials have been conducted using ECFCs isolated from bone marrow or peripheral blood for the treatment of patients with lower-extremity ischemia. [42, 43]. Additionally, it has been reported that there is a relationship between cell survival, which is a limitation of cell transplantation in the body, and treatment of lower extremity ischemia [44–47]. We also showed that using spheroid cultures rather than adherent cultures to increase MSC survival and paracrine effects would increase the migration and tube formation of ECFCs.

MSCs and ECFCs are promising therapeutics for ischemic tissue regeneration. In this study, we demonstrated that transplantation of Hybrid^3D^, composed of MSCs and ECFCs, promoted blood perfusion, tissue repair, and angiogenesis in ischemic hindlimbs. Co-transplantation of ECFCs with MSCs has been reported to be beneficial in treating steroid-induced osteonecrosis of the femoral head and repairing bone and dental pulp tissues by promoting angiogenesis [48–50]. Furthermore, co-transplantation of MSCs with ECFCs has been reported to improve cardiac function in a myocardial infarction mouse model [51]. Co-cultured spheroids of human periodontal ligament MSCs and endothelial cells enhance periodontal tissue regeneration [33]. Injection of hybrid 3D spheroids composed of podocytes, MSCs, and endothelial cells into the renal cortex improved kidney function [52]. These results support the superior function of Hybrid^3D^ in in vivo angiogenesis and the regeneration of ischemic limbs. From these results, we suggest that hybrid spheroids containing MSCs can serve as carriers for co-transplantation of cell therapeutics, which have poor in vivo engraftment efficiency, such as ECFCs.

In this study, we demonstrated that Hybrid^3D^-derived conditioned medium promoted migration, tube formation, and proliferation of ECFCs in vitro, suggesting a paracrine angiogenesis activation. MSCs have been shown to improve the therapeutic potential of ECFCs in ischemic diseases [53–55]. We found that MSC^3D^ secreted various pro-angiogenic factors, including ENA-78, VEGF, MCP-3, IL-6, and PlGF. Increased expression of angiogenesis-promoting cytokines, including angiogenin, ANGPT2, VEGF, FGF-2, HGF, and IGF-1, has been reported in MSC spheroids [56, 57]. PlGF is an angiogenic factor that belongs to the VEGF family and activates VEGFR1 [58]. ENA-78 (epithelial neutrophil-activating peptide 78), a CXC chemokine, promotes angiogenesis and inflammation [59]. Moreover, ECFC^3D^ secretes increased levels of IL-6, PlGF, IL-8, TIMP-2, TIMP-1, and ANG compared to ECFC^2D^. Compared with MSC^3D^ and ECFC^3D^, Hybrid^3D^ secreted higher levels of PDGF-BB and ANGPT2 which could be mainly detected in the conditioned medium of ECFC^3D^, suggesting that the improved survival of ECFCs in Hybrid^3D^ may be attributed to the increased levels of these proteins. ANGPT2 activates endothelial cells and exerts a potent angiogenic action in the presence of VEGFA [60]. PDGF-BB is required for vessel maturation by increasing the recruitment and differentiation of endothelial cells [61]. ECFCs stimulate the expression of connexin 43 and integrin alpha-5 and the secretion of healing-associated molecules in MSCs, whereas MSCs prompt the organization of ECFCs into vascular networks, suggesting that the crosstalk between MSCs and ECFCs augments the therapeutic properties of MSCs and enhances the angiogenic properties of ECFCs [51]. Thus, it is likely that growth factors and cytokines secreted by Hybrid^3D^ are essential for regulating ECFC proliferation and migration.

In summary, our findings suggest that hybrid spheroids of MSCs and ECFCs are useful for angiogenesis and regeneration in lower-extremity ischemic diseases. Moreover, the hybrid spheroids enhanced the engraftment of co-transplanted ECFCs, suggesting that MSCs promote in vivo survival and angiogenic activities of ECFCs. Clarification of the intercellular crosstalk between MSCs and ECFCs within hybrid^3D^ will be needed for understanding the molecular mechanism associated with the hybrid^3D^-mediated therapy of ischemic diseases.

## Conclusions

The hybrid spheroids composed of MSCs and ECFCs exhibited enhanced therapeutic effects in hindlimb ischemia animal model. Spheroids from MSCs, but not from ECFCs, exhibited prolonged in vivo survival compared with adherent cultured cells. The hybrid spheroids increase angiogenesis through enhancing the engraftment of co-transplanted ECFCs in vivo*.* The present study suggests that hybrid spheroids of MSCs and ECFCs exhibit enhanced therapeutic efficiency and pro-angiogenic activities by enhancing the engraftment of transplanted cells.

## Supplementary Information

**Additional file 1**. Supplementary Figures 1 and 2.

## Data Availability

The data underlying this article will be shared on reasonable request to the corresponding author.

## Abbreviations

MSCs: Mesenchymal stem cells
ECFCs: Endothelial colony-forming cells
PAD: Peripheral arterial diseases
EGM-2: Endothelial basal medium-2
PBS: Phosphate buffered saline
LDPI: Laser doppler perfusion imaging
VEGF: Vascular endothelial growth factor
MSC^3D^: MSC spheroids
ECFC^3D^: ECFC spheroids
Hybrid^3D^: Co-culture of MSCs and ECFCs in the ultra-low attachment dish forms mixed spheroids
MSC^2D^: Two-dimensional cultured MSCs
ECFC^2D^: Two-dimensional cultured ECFCs
Hybrid^2D^: Mixture of MSC^2D^ ad ECFC^2D^
PlGF: Placental growth factor
ENA-78: Endothelial neutrophil-activating peptide 78
