# Supplementary material for: Hybrid spheroids containing mesenchymal stem cells promote therapeutic angiogenesis by increasing engraftment of co-transplanted endothelial colony-forming cells in vivo
Source: Stem Cell Res Ther. 2023 Aug 2;14:193. doi: 10.1186/s13287-023-03435-z (PMC10394850; doi:10.1186/s13287-023-03435-z)
Supplement: Supplementary file 1 — Additional file 1. Supplementary Figures 1 and 2. [file 13287_2023_3435_MOESM1_ESM.docx]

**Hybrid Spheroids Containing Mesenchymal Stem Cells Promote Therapeutic Angiogenesis by Increasing Engraftment of Co-transplanted Endothelial Colony-Forming Cells *In Vivo***

Young Cheol Song^1*^, Gyu Tae Park^1*^, Hye Ji Moon^1^, Eun-Bae Choi^1^, Mi-Ju Lim^1^, Jung Won Yoon ^1^, Nayeon Lee^1,2^, Sang Mo Kwon^1^, Byung-Joo Lee^3^, and Jae Ho Kim^1,2, ¶^

^1^Department of Physiology, College of Medicine, Pusan National University, Yangsan 50612, Gyeongsangnam-do, Republic of Korea; ^2^Convergence Stem Cell Research Center, Medical Research Institute, Pusan National University, Yangsan 50612, Gyeongsangnam-do, Republic of Korea; ^3^Department of Otorhinolaryngology-Head and Neck Surgery, College of Medicine, Pusan National University and Biomedical Research Institute, Pusan National University Hospital, Busan 49241, Korea.

^¶^**Corresponding Author:** Jae Ho Kim, Ph.D., Department of Physiology, Pusan National University School of Medicine, Yangsan 50612, Gyeongsangnam-do, Republic of Korea. Tel.: 82-51-510-8073; Fax: 82-51-510-8076; E-mail: [jhkimst@pusan.ac.kr](mailto:jhkimst@pusan.ac.kr)

*Equally contributed authors

**Supporting Information**





**Supplementary Figure 1. Characterization of surface markers of MSCs and ECFCs.**

Morphology of MSCs (A) and ECFCs (B). Flow cytometric analysis of MSCs (C) and ECFCs (D). The expression of MSC-positive markers (CD166 and CD44) and ECFC-positive markers (CD34 and CD31) in MSCs and ECFCs was measured by FACS analysis.


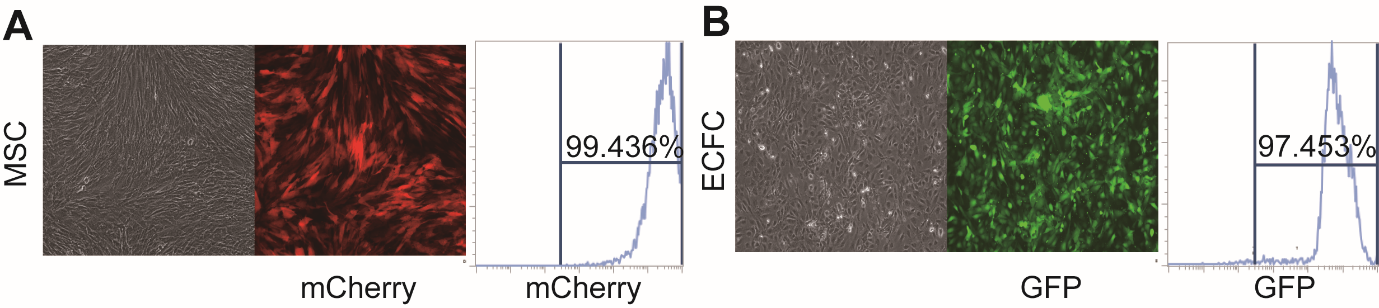


**Supplementary Figure 2. Establishment of mCherry-expressing MSCs and GFP-expressing ECFCs.**

(A) Left panel: The phase contrast and fluorescent images of MSC-mCherry. Right panel: FACS analysis of MSC-mCherry. The percentage of mCherry-positive cells are indicated. (B) Left panel: The phase contrast and fluorescent images of ECFC-GFP. Right panel: FACS analysis of ECFC-GFP. The percentage of GFP-positive cells are indicated.
